# The Role of Stress and Genital Immunity in Sexual Trauma and HIV Susceptibility Among Adolescent Girls and Adult Women (The THRIVE Study): Protocol for a Longitudinal Case-Control Study

**DOI:** 10.2196/18190

**Published:** 2020-12-07

**Authors:** Jamila K Stockman, Katherine M Anderson, Maile Y Karris, Constance A Benson, Kiyomi Tsuyuki, Douglas A Granger, Akilah Weber, Mimi Ghosh

**Affiliations:** 1 Division of Infectious Diseases and Global Public Health Department of Medicine University of California, San Diego La Jolla, CA United States; 2 Institute for Interdisciplinary Salivary Bioscience Research University of California, Irvine Irvine, CA United States; 3 School of Nursing Bloomberg School of Public Health Johns Hopkins University School of Medicine Baltimore, MD United States; 4 Rady Children's Hospital San Diego San Diego, CA United States; 5 Department of Obstetrics and Gynecology University of California San Diego Health San Diego, CA United States; 6 Department of Epidemiology Milken Institute School of Public Health The George Washington University Washington, DC United States

**Keywords:** sexual trauma, sexual violence, forced sex, HIV risk, stress, genital immunity, adolescent girls, adult women, United States, case-control, longitudinal

## Abstract

**Background:**

The relationship between sexual violence and HIV risk has been extensively documented through social and behavioral research; however, the underlying biological mechanisms are poorly understood.

**Objective:**

The purpose of the THRIVE (Trauma and HIV Risk: Investigating Stress and the Immune Disruption of the Vaginal Environment) Study is to examine the impact of sexual trauma due to sexual violence on HIV susceptibility through dysregulation of soluble inflammatory and anti-inflammatory and anti-HIV biomarkers in the female genital tract and dysregulation of the hypothalamic-pituitary-adrenal axis among adolescent girls and adult women.

**Methods:**

The THRIVE Study is a longitudinal case-control study conducted in San Diego, CA, among a racially diverse sample. Cases are adolescent girls (aged 14-19 years) or adult women (aged 20-45 years) who have experienced forced vaginal penetration by a phallus perpetrated by a man within the past 15 days. Controls are adolescent girls or adult women who have engaged in consensual vaginal sex with a man within the past 15 days. At baseline and 1- and 3-month follow-up study visits, participants undergo a urine-based pregnancy test; venipuncture blood draw for HIV, C-reactive protein, adrenocorticotropic hormone, and progesterone testing; a 45-min interviewer-administered computer survey; and cervicovaginal lavage to measure proinflammatory and anti-inflammatory and anti-HIV soluble immune biomarkers. After each study visit, participants self-collect saliva specimens (upon waking, 30 min after waking, and 45 min after waking) at home for 3 consecutive days, which are later assayed for cortisol and dehydroepiandrosterone sulfate. Participants receive compensation at each study visit and for the return of saliva specimens, and a list of local medical and support services. Study procedures use trauma-informed care methods, given the sensitive nature of the study and enrollment of women in the acute phase after sexual trauma. All research staff and investigators adhere to ethical principles and guidelines in the conduct of research activities. Data will be analyzed for descriptive and inferential analyses.

**Results:**

The recruitment of participants is ongoing. The publication of the first results is expected by late 2021.

**Conclusions:**

The THRIVE Study will provide foundational knowledge on how sexual trauma due to sexual violence increases susceptibility to HIV acquisition via alterations in cervicovaginal immune regulation and the psychobiology of the stress responses. These findings will inform future research on mechanistic models of in vitro and in vivo injury and cervicovaginal wound healing processes, which may lead to the development of nonvaccine biomedical HIV prevention products for girls and women.

**International Registered Report Identifier (IRRID):**

DERR1-10.2196/18190

## Introduction

### Background

In the United States, racial and ethnic- and age-related disparities in the intersecting epidemics of sexual violence and HIV are paramount [1-4]. The type of sexual violence most often implicated as a risk factor for HIV acquisition is forced sex, defined as unwanted or nonconsensual penetrative sex that occurs through the use of violence, physical force, or threats thereof [1,5]. Although national statistics highlight equivalent lifetime prevalence rates of forced sex (a form of sexual violence) for Black and White women (21%) and lower rates among Latinx women (14%) [5], multiple US community-based studies have documented a forced sex lifetime prevalence rate as high as 38%-54% for Black women [6-8] and 21%-38% for Latinx women [9-11]. More severe forms of sexual trauma due to forced sex or sexual violence have also been reported among Black and Latinx women [12,13]. Among women who have experienced rape, 40% were raped before the age of 18 years and 79% were raped before the age of 25 years [5]. Specific to HIV infection, women account for 20% of all new HIV infections in the United States, of which 22% occur in adolescent girls and adult women between the ages of 13 and 24 years [14]. Women of color account for most of the incident and prevalent HIV infections and HIV-related deaths among women in the United States [14]. The likelihood of a woman being diagnosed as having HIV in her lifetime is significantly higher for Black (1 in 54) and Latinx (1 in 256) women than for White women (1 in 941) [15].

**Figure 1:**
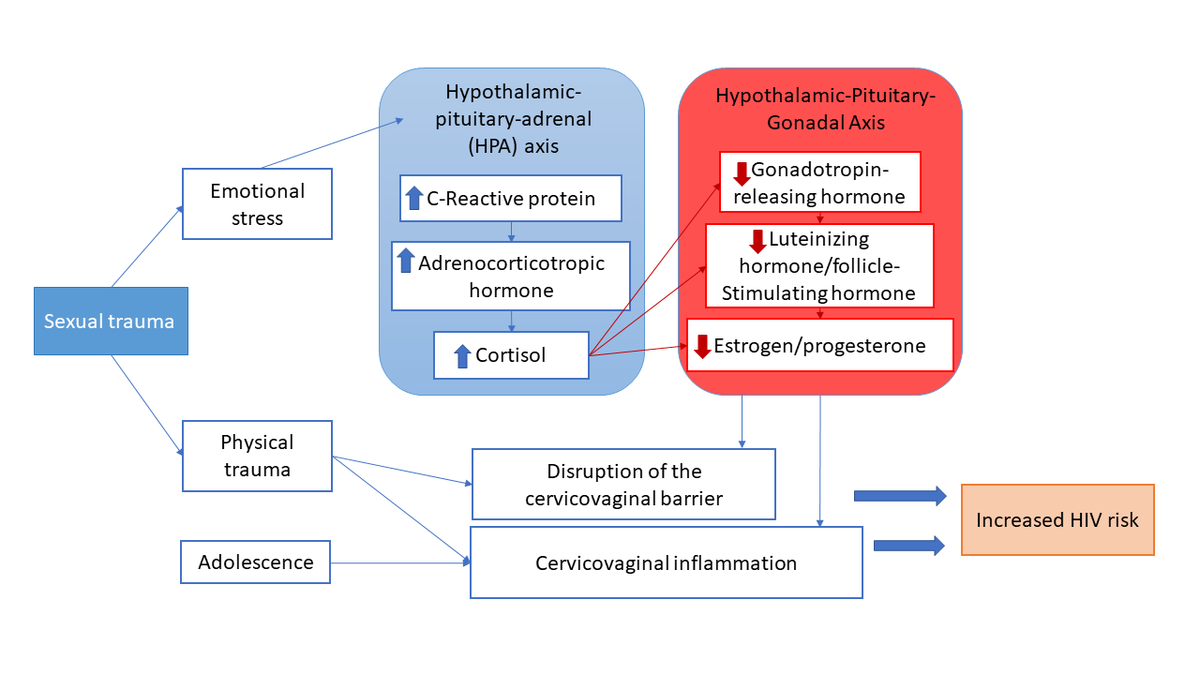
Conceptual model for the THRIVE Study. THRIVE: Trauma and HIV Risk: Investigating Stress and the Immune Disruption of the Vaginal Environment.

The sexual- and drug-related behavioral pathways linking sexual violence and HIV susceptibility have been extensively documented [1,2,16]. However, the biological mechanisms driving increased HIV susceptibility following a forced sex incident are understudied, despite documentation of plausible pathways [1,17-19]. A potential pathway is that following trauma due to sexual violence, there is immune dysregulation in the female genital tract (FGT) secretions (ie, cervicovaginal inflammation) that may increase HIV susceptibility (Figure 1) [19-21]. These changes may be due to rough or violent, nonconsensual vaginal or anal intercourse; genital injuries, such as abrasions, tears, and microlesions in the epithelium of the vagina, cervix, or anal regions; sexually transmitted infections (STIs) that cause cell death; mucosal inflammation; and local activation of CD4+ T cells, which may increase the likelihood of HIV transmission [17,19,22]. The production of proinflammatory cytokines as a result of sexual trauma may be disrupted and delay wound repair [18], further compromising the vaginal and anal epithelium [19]. Adolescents may face compounded risk as a result of the immaturity of the FGT and incomplete development of the vaginal, ectocervical, and cervical epithelia [23].

Moreover, as a result of the psychological consequences of intense sexual trauma, individual differences in the effects on the reactivity and regulation of the hypothalamic-pituitary-adrenal (HPA) axis have the potential to affect the innate and adaptive immune system in the FGT [24], further influencing HIV susceptibility (Figure 1) [25-27]. High and variable levels of HPA axis activation also have consequences for the secretion of estrogen and progesterone through effects on the gonadotropin-releasing hormone [28]. The pubertal stage, shifts in emotional lability, increased levels of hormones, and rapid brain development in adolescents render them particularly susceptible to the adverse effects of stress [28].

Sexual trauma may affect the normal stress response (1) immediately via the sexual trauma incident, (2) by the physical and psychological violence that accompanies sexual trauma, and (3) via continued retraumatization and re-experiencing of the sexual trauma after the incident [29,30]. HPA dysregulation has been documented in acute and chronic sexual abuse victims [25,31,32], and it is suggested that alterations in the HPA axis functioning may occur relatively proximate to the traumatic event and correlate with hyperarousal symptoms [26]. Trauma-induced changes in the regulation of the HPA axis may manifest for years after the original trauma or stressor [33]. Chronic and sustained activation of the HPA axis in response to stress can lead to abnormal levels of reproductive hormones, which also has the potential to influence the immune system [17,34]. Specifically, traumatic stress promotes a proinflammatory profile [35], which may increase susceptibility to HIV, either directly by cortisol binding to receptors on immune cells or indirectly by disrupting the regulation of cytokines [18,34].

The pronounced racial and ethnic disparities in sexual violence and HIV highlight the importance of considering potential racial and ethnic group differences in the underlying biological mechanisms. Women of color experience chronic stress because of social and cultural factors (eg, discrimination, medical mistrust, and immigration status) [36,37]. In addition, low socioeconomic status and ethnic minority status are associated with increased psychosocial stress and elevated circulating concentrations of C-reactive protein (CRP), a marker of chronic inflammation, which may indicate preexisting immune dysregulation that could be exacerbated by sexual trauma [37,38]. Furthermore, there are observed differences between Black and White or Latinx women with regard to genes associated with inflammation and antimicrobial immunity [39].

Given the lack of understanding of the biological pathways contributing to the relationship between sexual violence and increased HIV susceptibility, it is critical to explore the potential biological mechanisms at play. This is particularly important given the increased risk of sexual violence and HIV independently among adolescents and women of color, and the possible compounding effect of biology with society and culture.

### Objectives

The primary aim of the THRIVE Study is to assess the impact of sexual trauma on the FGT immunity in adolescent girls and adult women by evaluating (1) the disruption of genital immune biomarkers after sexual trauma and (2) the functional loss of anti-HIV immunity against laboratory-adapted and transmitter/founder (T/F) strains of HIV in genital secretions after sexual trauma. The secondary aim is to assess the impact of sexual trauma on HPA axis dysregulation in adolescent girls and adult women by evaluating (1) correlations between the dysregulated HPA axis and genital immune biomarkers and (2) correlations between the dysregulated HPA axis and functional loss of anti-HIV immunity in genital secretions. Finally, the third aim is to examine whether risk factors linked to sexual trauma, including sexual risk behaviors, substance use, and mental health, influence HPA axis dysregulation and FGT immunity in adolescent girls and adult women after sexual trauma.

## Methods

### Study Design

The THRIVE (Trauma and HIV Risk: Investigating Stress and the Immune Disruption of the Vaginal Environment) Study is a longitudinal case-control study among girls and women aged 14-45 years, with baseline and 1- and 3-month follow-up study visits. Cases include adolescent girls aged 14-19 years (n=30) and adult women aged 20-45 years (n=30) who have experienced vaginal sexual trauma within the past 15 days. Controls include adolescent girls aged 14-19 years (n=30) and adult women aged 20-45 years (n=30) who have engaged in consensual sex within the past 15 days. The goal is to recruit an ethnically diverse (ie, Black, White, Latinx, and Asian) sample of English- or Spanish-speaking adolescent girls and adult women in each study group.

### Study Population

Inclusion criteria for the THRIVE Study are (1) biologically female; (2) HIV negative at baseline; (3) either aged 14-19 (adolescent girls) or 20-45 (adult women); (4) having experienced forced vaginal penetration by a phallus, perpetrated by a man, within the past 15 days (cases) or having engaged in consensual sex via vaginal penetration within the past 15 days (controls); and (5) ability to provide informed consent (if aged 18 years and older), or ability to provide informed assent (if under the age of 18 years). Exclusion criteria are (1) currently pregnant or breastfeeding, (2) use of douches or other vaginal products within 15 days before baseline, (3) cognitive impairment that would limit participation in study procedures, and (4) for cases, consensual vaginal penetration between the experience of forced vaginal penetration and baseline study visit.

### Study Procedures

#### Recruitment and Screening

Recruitment methods for the THRIVE Study include a three-pronged approach to facilitate the sampling of an ethnically diverse study population. First, we conduct passive recruitment of hard-to-reach populations (ie, adolescents and women of color) through targeted flyer distribution, using a recruitment theme of empowerment and capitalizing upon current movements in women’s rights. Canvassing activities focus on neighborhoods in San Diego County, where more than 10% of the population is Black or Latinx, and areas with a high incidence of sexual violence. Second, we use rigorous outreach methods for passive recruitment through print and social media, including advertisements in local newspapers and a robust presence on Facebook and Instagram, an accessible branded website, and the creation of spaces within social media for sharing, advocacy organizing, and engaging with the THRIVE Study. Finally, we partner with ancillary support agencies (eg, rape crisis centers, mental health providers, community centers, and housing services), community centers, and health clinics to passively recruit through flyer distribution and to actively recruit or refer via agency employees (eg, providers, social workers, case managers, and advocates) who have direct interaction with potential participants. Potential participants who are interested in the THRIVE Study are given the study contact information to call or email study staff or provide verbal permission for collaborating organizations to provide their contact information to the THRIVE Study staff.

Girls and women interested in participating in the study are either screened for eligibility over the phone by study staff or complete a 5-min web-based screening survey, which is then reviewed by study staff for eligibility. Participants provide digital or verbal consent to screen before responding to the screening questions. Screening questions include demographics, sexual violence history, consensual sexual behavior history, pregnancy and breastfeeding status, and ability and willingness to participate in at-home saliva sample collection following each study visit. After completion of the screener, girls and women are notified of their eligibility status. Eligible and interested participants are scheduled to attend the baseline study visit. Ineligible participants are informed that their eligibility status may change, and if interested, they are invited to rescreen in the future.

#### Study Visits

At the baseline visit (duration of 1.5-3 hours), participants provide written informed consent (adult women) or assent (adolescent girls) and complete study activities. At baseline and 1- and 3-month postbaseline visits, participants complete the study activities, receive US $50, and are provided with a list of local health and support resources and transportation assistance. Following each study visit, participants complete 3 days of at-home self-collection of saliva specimens. Participants have the choice to deliver the saliva specimens or have the study staff retrieve them. Following retrieval of each of the saliva specimen collection periods, participants receive an additional US $35.

At each study visit, participants undergo (1) a urine test, (2) venipuncture blood sample collection, (3) an interviewer-administered quantitative survey, and (4) a cervicovaginal examination. Biological biomarkers and survey measures are listed in detail in Tables 1 and 2, respectively. Following each study visit, participants self-collect saliva specimens at home for 3 consecutive days. All study staff are trained on research methods, data collection, confidentiality, trauma-informed care, safety and security protocols, protection of human subjects, and mental health first aid. Trained and licensed clinical staff collect all biological specimens and interpret the test results.

**Table 1:** Biological sample biomarkers.

Biological sample and biomarker		Test or method	Source of the protocol
**Dipstick pregnancy test**			
	hCG^a^	Sure-Vue	Manufacturer
**Venipuncture blood draw**			
	HIV 1/2 antibody and p24 antigen	CMIA^b^	UCSD^c^ Health Clinical Laboratories
	Progesterone	ECLIA^d^	UCSD Health Clinical Laboratories
	C-reactive protein	Latex immunoturbidimetry	UCSD Health Clinical Laboratories
	ACTH^e^	ECLIA	ARUP^f^ Laboratories
**Vaginal swabs**			
	Candida, Gardnerella, and Trichomonas	Affirm VPIII Ambient Temperature Transport System	Manufacturer
	*Chlamydia trachomatis* (Chlamydia) and *Neisseria gonorrhoeae* (Gonorrhea)	Cobas CT/NG^g^	Manufacturer
**Cervicovaginal lavage**			
	IL^h^-1α, IL-1β, IL-6, IL-8, TNF-α^i^, MIP3α^j^, SLPI^k^, elafin, and β-defensin 2	ELISA^l^ (R&D Systems and PeproTech)	Ghosh et al [40] and Lahey et al [41]
**Passive drool saliva samples**			
	Cortisol and DHEA-S^m^	ELISA (SalivaBio and Salimetrics)	Granger et al [42] and Wilde et al [43]

^a^hCG: human chorionic gonadotropin.

^b^CMIA: chemiluminescent microparticle immunoassay.

^c^UCSD: University of California, San Diego.

^d^ECLIA: electrochemiluminescence immunoassay.

^e^ACTH: adrenocorticotropic hormone.

^f^ARUP: Associated Regional and University Pathologists.

^g^CT/NG: *Chlamydia trachomatis* or *Neisseria gonorrhoeae*.

^h^IL: interleukin.

^i^TNF-α: tumor necrosis factor-alpha.

^j^MIP3α: macrophage inflammatory protein-3α.

^k^SLPI: secretory leukocyte peptidase inhibitor.

^l^ELISA: enzyme-linked immunosorbent assay.

^m^DHEA-S: dehydroepiandrosterone sulfate.

**Table 2:** Exposure and outcome measures: quantitative survey.

Variable group and measures		Source of measures
**Demographic characteristics^a^**		
	Race and ethnicity	N/A^b^
	Nativity	N/A
	Sexual orientation	N/A
	Marital status	N/A
	Number of children	N/A
	Educational attainment	N/A
	Student status	N/A
	Employment status	N/A
	Living situation	N/A
	Socioeconomic status	N/A
**Mental health history**		
	PTSD^c^ (preassault)^d^	PC-PTSD^e^ [44]
	Depression (preassault)^d^	CES-D^f^ [45]
	Perceived stress (preassault)^d^	PSS^g^ [46]
	Resilience	Connor-Davidson Resilience Scale [47]
	Lifetime of traumatic events^a^	Lifetime Events Checklist [48]
	Previous experiences with battering	Women’s Experiences with Battering Scale [49]
**Sociocultural factors^a^**		
	Gender role beliefs	Gender Role Beliefs Scale [50]
	Sexual relationship power	Sexual Relationship Power Scale [51]
	Discrimination	Extended Everyday Discrimination Scale [52]
	Medical mistrust	GBMMS^h^ [53]
	Law enforcement mistrust	Adapted from GBMMS
**Medical history**		
	Current symptoms	Created for the THRIVE^i^ Study
	Diagnosis history^a^	N/A
	Use of medications and substances	N/A
**Gynecologic and reproductive history**		
	Menstrual history	Created for the THRIVE Study
	STI^j^ and HIV testing history	N/A
	STI diagnosis and treatment history	N/A
	Pregnancy history	National Survey of Family Growth and CDC^k^ Reproductive Health Assessment Questionnaire for Conflict-Affected Women [54]
	Pregnancy complication history	National Survey of Family Growth and CDC Reproductive Health Assessment Questionnaire for Conflict-Affected Women [54]
	Lifetime^a^, recent^a^, and current contraceptive use	National Survey of Family Growth and CDC Reproductive Health Assessment Questionnaire for Conflict-Affected Women [54]
**Sexual behavior history**		
	Sexual debut^a^: age, contraceptive or protective method use, substance use, coercive or forced experience	Created for the THRIVE Study
	Recent sexual behavior	N/A
	Lifetime^a^ and recent sexual partners	N/A
	Concurrent sexual partners	N/A
	Lifetime^a^ and recent sexually violent experiences	Created for the THRIVE Study
**Current and former substance use**		
	Lifetime^a^ and recent substance use before sexual activity	N/A
	Recent alcohol use behaviors	10-item Alcohol Use Disorders Identification Test [55]
	Lifetime^a^ and recent drug use behaviors	NIDA^l^ Quick Screen and NIDA-Modified Alcohol, Smoking and Substance Involvement Screening Test [56]
	Lifetime^a^ and recent methods of drug administration	Created for the THRIVE Study
**Sexual assault^a,d^**		
	Perpetrator use of alcohol and drugs	Created for the THRIVE Study
	Survivor use of alcohol and drugs (consensual and nonconsensual)	Created for the THRIVE Study
**Postassault change^a,d^**		
	Substance use postassault	Created for the THRIVE Study
	PTSD postassault	PC-PTSD [44]
	Depression postassault	CES-D [45]
	Perceived stress postassault	PSS [46]
**Referral assessment**		
	Assessment of suicidal ideation	Suicide Behaviors Questionnaire-Revised [57]
	Assessment of the likelihood of partner homicide	Danger Assessment [58]

^a^Denotes measures collected at baseline (month 0) only.

^b^N/A: not applicable.

^c^PTSD: posttraumatic stress disorder.

^d^Denotes baseline measures asked of case participants only.

^e^PC-PTSD: Primary Care PTSD Screen.

^f^CES-D: Center for Epidemiologic Studies Depression Scale.

^g^PSS: Perceived Stress Scale.

^h^GBMMS: Group-Based Medical Mistrust Scale.

^i^THRIVE: Trauma and HIV Risk: Investigating Stress and the Immune Disruption of the Vaginal Environment.

^j^STI: sexually transmitted infection.

^k^CDC: Centers for Disease Control and Prevention.

^l^NIDA: National Institute on Drug Abuse.

##### Urine Test

Every participant is asked to provide a urine sample. The urine sample is collected by clinical laboratory staff, and a urine dipstick test (Sure-Vue, Fisher Scientific) is used to test for pregnancy via assessment of human chorionic gonadotropin hormone (Table 1). Participants with a positive test result at baseline are provided with counseling, education on pregnancy options (eg, parenting, abortion, or adoption), and referrals by the study physician or nurse practitioner, and then they are administratively withdrawn from the study. Participants testing positive at a follow-up study visit are provided similar items but are not administratively withdrawn from the study.

##### Venipuncture Blood Sample Collection

Participants undergo low-volume peripheral blood draws performed by clinical laboratory staff. Blood samples are processed by clinical laboratory staff and sent to the University of California, San Diego (UCSD) Health Clinical Laboratories for testing. All blood samples undergo assaying for serum CRP and progesterone using the immunoturbidimetric latex and chemiluminescence methodology, respectively (Table 1). When possible, study visits are scheduled to facilitate blood draws between 7 and 10 AM, the time frame in which adrenocorticotropic hormone (ACTH) levels peak. The additional sample is collected in an EDTA tube to be processed and transported to be tested for plasma ACTH at UCSD Health Clinical Laboratories.

##### HIV Testing

From the low-volume peripheral blood draw, a blood sample is collected, processed for transport, and transported to the UCSD Health Clinical Laboratories for HIV-1/2 antibody and p24 antigen testing, followed by HIV RNA testing if the initial test result is positive or inconclusive (Table 1). Participants who have a positive test result for HIV at baseline are provided with counseling, referrals, and linkage to HIV care by the study physician or nurse practitioner, after which they are administratively withdrawn from the study. Participants with a positive test result for HIV at a follow-up visit undergo the same process but are not administratively withdrawn from the study to capture HIV incidence.

##### Quantitative Survey

At each study visit, all participants complete a 45-min interviewer-administered computer survey using Research Electronic Data Capture (Vanderbilt University) hosted by the UCSD Altman Clinical and Translational Research Institute. Participants are shown visual analog response cards for measures with Likert response scales to reduce the participant burden. The quantitative survey includes the following 10 domains (Table 2): demographic characteristics, mental health, sociocultural factors, medical history, gynecologic and reproductive history, sexual behavior, substance use, sexual assault, postsexual assault, and referral assessment for suicide risk and potentially lethal relationships. Questions on sexual trauma and postsexual trauma experiences are only asked for case participants. Sexual trauma history questions assess perpetrator and participant use of alcohol and drugs during the incident of sexual violence that met the study inclusion criteria, both consensually and nonconsensually. The section on postsexual assault experiences assesses changes in behaviors and mental health in the time since participant’s experiences of sexual violence, including changes in substance use, posttraumatic stress disorder, depression, and perceived stress, and the participant’s use of formal and informal support services.

##### Cervicovaginal Examination

Following the completion of the survey, participants undergo a cervicovaginal examination performed by a female physician or nurse practitioner specialized in infectious diseases and women’s reproductive health. This examination includes a visual inspection of the cervicovaginal epithelium to assess current STI symptomology and the collection of vaginal swabs and cervicovaginal lavage (CVL) fluid (Table 1).

###### STI Testing

Vaginal swabs are collected by rotating a single-use specimen swab along the vaginal epithelium and are used to test for vaginal candida (yeast), gardnerella (bacterial vaginosis), trichomonas, chlamydia, and gonorrhea. For candida, gardnerella, and trichomonas, the Affirm VPIII Ambient Temperature Transport System is used to preserve the sample for testing. Chlamydia and gonorrhea nucleic acid amplification testing is performed using the Cobas CT/NG (*Chlamydia trachomatis* and *Neisseria gonorrhoeae*) testing system. All vaginal swabs are sent to the UCSD Health Clinical Laboratories for testing.

###### CVL

During the cervicovaginal examination, the study physician or nurse practitioner collects cervicovaginal cells using a disposable speculum lubricated with a water-based lubricant to dilate the cervicovaginal canal. The cervix and ectocervix are bathed with 5 mL of 0.9% saline solution. The saline solution is allowed to pool in the posterior fornix and then aspirated into a syringe. The study physician or nurse practitioner repeats this process 5 times to ensure adequate suspension of cervicovaginal epithelial cells. The CVL fluid is then centrifuged, the supernatant and pellet are separated, and both samples are frozen at −80°C until batch shipment to the George Washington University Milken Institute School of Public Health. Supernatants from CVL samples will be tested for proinflammatory (interleukin [IL]-1α, IL-1β, IL-6, IL-8, and tumor necrosis factor-alpha [TNF-α]) and anti-inflammatory and anti-HIV (secretory leukocyte peptidase inhibitor [SLPI], elafin, β-defensin 2, and macrophage inflammatory protein-3α [MIP3α]) soluble immune biomarkers. These biomarkers will be quantified using enzyme-linked immunosorbent assays from R&D Systems and PeproTech. The functional anti-HIV activity of CVL will be determined using the TZM-bl assay.

##### Saliva Specimen At-Home Self-Collection

Following the completion of each study visit, participants are instructed on the saliva specimen at-home self-collection process. Saliva is used to measure cortisol and dehydroepiandrosterone sulfate (DHEA-S; Table 1). Participants use a prepackaged kit to self-collect saliva samples at home 3 times a day (upon waking, 30 min after waking, and 45 min after waking) for 3 days following each study visit. The prepackaged kit contains instructions, 3 snack-size bags with cryovial tubes (for each collection day), SalivaBio saliva collection aids, and a log form. Using the passive drool method, pooling saliva at the bottom of the mouth, and then easing it into the collection device directly, participants self-collect at least 1 mL of saliva in each cryovial tube. Samples are stored in a −20°C freezer (or general home freezer) immediately following collection. The log form is used for participants to self-report any deviation from their wake-up schedule, actual collection times, stressors, or unexpected activities (eg, brushing teeth, smoking, and eating). To facilitate ease of sampling and increased adherence, this protocol includes a thorough explanation of collection procedures at each study visit; scheduling of wake-up times; reminder text messages and phone calls; supply of a study mobile phone if the participant does not have a phone, cannot receive messages, or feels unsafe or uncomfortable receiving study messages to her personal mobile phone; and pick up of samples by study staff. The saliva collection protocol is based on previously published recommendations for home-based saliva sample collection, participant preparation, and sample handling [26,42]. On the third day of collection, the study staff travels to participants’ residence to pick up the saliva samples. Samples are then batch shipped (using dry ice) to the University of California Irvine Institute for Interdisciplinary Salivary Bioscience Research, where they are stored frozen at −80 °C until the day of the assay. Samples are assayed in duplicate using commercially available immunoassays for cortisol and DHEA-S, specifically designed for use with saliva, according to the manufacturer’s recommended protocols (Salimetrics). The average of the duplicate assays is used in the statistical analysis. Cortisol is measured in micrograms per deciliters and DHEA-S in picogram per milliliters.

Using the waking, 30-min postwaking, and 45-min postwaking samples, the daily cortisol awakening response (CAR) is calculated; using the area under the curve increase to calculate the CAR accounts for the change over time in cortisol with respect to baseline, which is the waking sample in this study. Next, we compute the mean between the 3 days for an average CAR for each participant. The average CAR will be used for the study analyses [59].

##### Safety Protocol

Due to the highly sensitive nature of this study and the acute phase following sexual trauma during which cases are enrolled in the study, the THRIVE Study has taken additional measures to develop and implement a safety protocol. This protocol outlines the information on how to respond to sensitive circumstances that may arise during study appointments, including mandated reporting of child abuse, expected and unexpected adverse events, including extreme emotional distress and suicidal ideation, and identification of a participant in an abusive relationship or at risk of homicide by a violent partner. Within the safety protocol, the staff is trained in ways to address participant sensitivity and trauma and to explain our role as mandated reporters of any incidents of childhood abuse reported during participation in the study. In addition, the safety protocol outlines resources that are distributed to all participants, which include ancillary services for domestic violence (eg, shelter and counseling), legal assistance, substance use, and medical care. Finally, as part of the safety protocol, all women receive counseling on community awareness of sexual violence and partner violence as well as resources to share with their community.

### Data Management and Quality Assurance

Several precautions are taken with participant data to protect confidentiality. All participants are assigned a numeric personal identification number, which is used as a reference to the participant on all study data to delink the study databases from personal identifying data. Only a few study personnel have access to deidentified project files and databases, and the lowest level of access acceptable for a staff member’s role is only granted after full ethical training and upon the principal investigator’s approval. All study materials on paper containing participant information (eg, contact information sheet and signed consent forms) are stored in a locked cabinet in a locked office, accessible by the project coordinator and principal investigators, and within a building with restricted access.

Quantitative deidentified screening data are downloaded on a weekly basis and distributed graphically and in a tabular format to the project coordinator and principal investigators to monitor recruitment, screening, and enrollment of participants. Deidentified biological samples from blood and vaginal swabs are sent to the laboratories at UCSD for assaying or testing of CRP, progesterone, ACTH, HIV, STIs, yeast infections, and bacterial vaginosis; CVL samples are sent to the George Washington University for assaying of proinflammatory (IL-1α, IL-1β, IL-6, IL-8, and TNF-α) and anti-inflammatory and anti-HIV (SLPI, elafin, β-defensin 2, and MIP3α) soluble immune biomarkers; and saliva specimens are transported to the University of California, Irvine, for assaying of cortisol and DHEA-S. Test results from UCSD are delivered deidentified on paper and directly entered into REDCap, whereas test results from the George Washington University and the University of California Irvine are sent deidentified via email to UCSD and securely stored in the OneDrive THRIVE Study folder. Biological specimens are stored for 6 months to allow for retesting, if necessary, and then disposed of as medical hazard waste.

### Ethical Conduct of Human Subjects Research Approval

The THRIVE Study has been approved by the UCSD Human Research Protection Program (HRPP; UCSD HRPP Project #181898). The institutional review boards at the George Washington University and the University of California, Irvine, approved reliance agreements from the institutional review board at UCSD. Before working with the THRIVE Study, all UCSD staff received training in the ethical conduct of human subjects research, compliance, and data management via a collaborative institutional training initiative for biomedical research and Health Insurance Portability and Accountability Act (HIPAA). Women 18 years and older who are interested in participating in the THRIVE Study are asked to sign an informed consent form before participating, whereas adolescent girls younger than 18 years are asked to sign an informed assent form. A waiver of parental assent allows for the protection of privacy for young girls, given the sensitive topic of sexual trauma and sexual intercourse, and is in accordance with the UCSD HRPP’s recommendations. In addition, it is in consonance with the waiver of parental assent implemented for adolescents receiving services at the rape crisis center, a collaborating recruitment site. All participants are asked to sign a HIPAA authorization form. Finally, a certificate of confidentiality for the THRIVE Study is automatically issued by the funding agency, the National Institutes of Health, to protect identifiable research information from forced disclosure (eg, substance use behaviors).

### Data and Safety Monitoring

The THRIVE Study uses a data and safety monitoring plan, which is detailed in a standard operating procedures manual as a reference for secure data collection, management, and monitoring procedures for all study staff. Refresher training sessions for staff are scheduled as needed. All data collected for the THRIVE Study are stored on a secure encrypted drive in a locked office within a secure, locked suite in a clinical research building. Participants’ identifying information is stored separately from numeric participant identification numbers. The linkage between identifying information and study data is maintained through Ripple [60], a HIPAA-compliant secure web application designed for the management of identifying information of participants. Ripple is used only for storing identifiable information of participants and not to capture other research data, ensuring the segregation of personally identifiable information and research data. Adverse events (eg, a breach in confidentiality or privacy and risk of serious and unanticipated harm) and serious adverse events (eg, hospitalization or death because of participation in study-related activities) are monitored by study staff. In the case of such an event, study staff make appropriate referrals to care for the participant, including, but not limited to, warm handoffs with the staff licensed therapist and local mental health resources. Immediately after adverse events, the staff is required to report to the principal investigator. The principal investigator reviews the adverse or serious adverse event and incident report and reports the event within 24 hours of its occurrence to the UCSD HRPP.

In addition, a consultant who is a nurse researcher and an international expert in the area of violence against women, with an emphasis on sexual and intimate partner violence and risk or lethality, is available for meetings and debriefing sessions with staff, as needed. Finally, a data and safety monitoring board convenes 2 times per year to review the progress of the THRIVE Study and assesses adherence to the data and safety monitoring plan. This board comprises a practicing infectious disease clinician, a biostatistician specializing in social epidemiology and HIV, and a public health researcher in HIV prevention and treatment. Recommendations are provided to the study team following each meeting.

### Analysis

#### Aim 1: Understand the Impact of Sexual Trauma on the FGT Immunity

Temporal trends in inflammatory and anti-inflammatory cytokines relative to postsexual trauma will be examined by producing frequency tables and bar graphs by time, age group, and case-control status. Measures of central tendencies and variability will be computed, and corresponding boxplots will be generated. Continuous variables that are not normally distributed will be log-transformed to reduce skew. We will use Student *t* tests and Wilcoxon signed-rank tests to compare continuous variables with *P* values lower than .05, considered significant. Spearman correlation coefficients will be computed to assess potential associations between continuous variables. We will use mixed (random effects) regression to examine the relationship between the case-control groups and the inflammatory and anti-inflammatory cytokines. Separate models will be constructed for adolescent girls and adult women. Models will account for known confounders, including the stage of the menstrual cycle, contraception use, and STI diagnosis after follow-up.

To evaluate the functional loss of anti-HIV immunity against laboratory-adapted and transmitted/founder strains of HIV in genital secretions of adolescent girls and adult women, we will use Kruskal-Wallis tests to compare CVL anti-HIV activity measured by percent HIV inhibition for selected strains, that is, laboratory-adapted R5-tropic virus and 3 mucosal-transmitted clade T/F viruses between case-control status by time. We will conduct two-group comparisons using Mann-Whitney *U* tests. This will be conducted for adolescent girls and adult women. We will also correlate the percent change in anti-HIV activity in CVL with alterations in each inflammatory and anti-inflammatory cytokine to postulate the mechanisms of immune dysfunction.

#### Aim 2: Assess the Impact of Sexual Trauma on the HPA Axis

The primary variables of interest to address this aim are CAR, DHEA-S to cortisol ratio, and ACTH levels. We will compute cortisol to DHEA-S ratios and follow the analytical methods outlined in aim 1 to examine the impact of sexual trauma on the HPA axis.

To determine the extent to which the HPA axis affects FGT immunity owing to sexual trauma, we will use Pearson correlation coefficient tests to test individual correlation statistics for pairs of the HPA axis (cortisol, ACTH) and FGT immunity variables (IL-1α, IL-1β, IL-6, IL-8, TNF-α, and %HIV inhibition). We will produce a correlation matrix to investigate the dependence between multiple variables at the same time, with correlation estimates measuring the direction and strength of the linear relationship among variables. We will also produce a symmetric scatterplot matrix of the variables as tested as correlations to visually observe the data. These results will be stratified by time and case-control status for adolescent girls and adult women.

#### Aim 3: Risk Factors Linked to Sexual Trauma Influence HPA Dysregulation and FGT Immunity

We will conduct comparisons between case-control status and independent variables using Pearson chi-square or Fisher exact test for dichotomous variables and *t* test and Wilcoxon rank-sum tests for continuous normally and non-normally distributed variables, respectively. We will use bivariate and multivariate logistic regression to examine associations between case-control status and survey variables (demographics, gynecologic and reproductive history, substance use, sexual behavior, and mental health status) at each time point.

To examine the relationship between (1) case-control status and the inflammatory and anti-inflammatory and anti-HIV mediators (ie, dysregulation of the FGT) and (2) case-control status and CAR and DHEA-S to cortisol ratio (ie, dysregulation of the central and peripheral HPA axis), mixed (random effects) regression models will be used. Separate models will be constructed for adolescent girls and adult women. Models will account for known confounders, including the stage of the menstrual cycle, contraception use, and STI diagnosis after follow-up. Given our small sample size and one of the benefits of using random effects regression models being that we can examine estimated changes for each subject, we will examine both subject-specific trends and population average trends.

### Sample Size Considerations

The goal of the THRIVE Study is to provide knowledge and data to facilitate future hypothesis-driven longitudinal research. As such, the THRIVE Study is an exploratory hypothesis-generating study rather than a hypothesis-testing study. The sample size was determined based on previous studies that have examined the immune microenvironment and wound healing in the female reproductive tract, with total sample sizes ranging between 18 and 77 women [18,40,41]. Given this range, we decided upon a sample size of 30 per case-control group in each age group (adolescent girls and adult women). Furthermore, our power calculation supported this decision. With 30 girls and 30 women in each group, there will be 82% of power to detect a standardized effect size for comparing with any 2 time point means of 0.75 with a two-sample *t* test and α=.05, 2-sided. As an example of detectable effect size, it is estimated that the standard deviation for SLPI, an anti-inflammatory and anti-HIV biomarker, is 26,000 units [41] so that mean differences of 19,500 and 13,520 units would correspond to a standardized effect size of 0.75 and 0.52, respectively. We expect attrition at the follow-up visits to be between 10% and 15%. However, because the proposed mixed and generalized estimated equations repeated measures approach uses all available data on each participant, these analyses should have very little loss of power because of attrition.

## Results

### Recruitment Timeline

Recruitment of potential participants began in January 2019, and enrollment of participants began in February 2019. Recruitment efforts began with control participants aged 18 years or older as the least sensitive population to be enrolled in the study. This allowed for iterative consideration of study procedures and participant burden and facilitated streamlining and honing of participant experience before enrollment of under 18 years and case populations. Adult case enrollment began in June 2019; however, active recruitment of cases did not begin until September 2019. Case participants enrolled before September 2019 contact the study based on recruitment materials targeting adult control participants. Recruitment of control and case participants under the age of 18 years began in November 2019. As of January 2020, recruitment and enrollment efforts have yielded screening of 557 potential participants and enrollment of 50 participants, including 8 case participants and 42 control participants.

### Screening and Enrollment

Of the 557 potential participants screened, 60.1% (335/557) had enough information to be classified into a study group: 74.3% (249/335) adult controls, 20.9% (70/335) adolescent controls, 3.3% (11/335) adult cases, and 1.5% (5/335) adolescent cases. Potential participants who screened and indicated a source were recruited through a variety of methods, including social media advertisements (335/468, 71.6%), paper flyers (89/468, 19.0%), referral by friends or family (26/468, 5.6%), and community newspaper advertisements (15/468, 3.2%). It is notable that most participants enrolled to date have been recruited through social media, including 63.1% (157/249) of adult controls, 60% (3/5) of adolescent cases, 64% (7/11) of adult cases, and 80% (56/70) of adolescent cases. With respect to cases, this may allow for access to individuals who may not seek services associated with traditional recruitment locations for recent survivors of sexual violence, such as emergency rooms or rape crisis centers. Of all participants screened, 45.1% (251/557) were classified as eligible, 30.0% (167/557) did not provide enough information to classify their eligibility, 19.0% (106/557) were ineligible, and 1.1% (6/557) declined to complete the screener. Of those eligible, 28.9% (73/253) were contacted and pursued for scheduling. Of those pursued for scheduling, 68% (50/73) were enrolled, 11% (8/73) cancelled before enrollment, and 18% (13/73) declined to enroll. Of the enrolled participants, 6% (3/50) of participants were administratively dropped because of positive STI at baseline. Of participants not administratively dropped or currently progressing through the study, 85% (35/41) have been retained to follow-up 1, and 76% (31/41) have been retained through study completion.

### Adult Control Participants

Of the control participants, 62% (26/42) are in the adult group (aged 20-45 years), with a mean age of 27.1 years (SD 7.6). Among adult control participants, 38% (10/26) of participants identify as Black or African American, 31% (8/26) identify as White, and 12% (3/26) as Asian. In addition, 31% (8/26) identify as Hispanic or Latinx (categories are not mutually exclusive). Regarding education, 38% (10/26) of adult control participants have graduated high school or received a General Educational Diploma (GED), 38% (10/26) have completed a bachelor’s or associate’s degree, and 65% (17/26) are current students. More than half of the adult control participants (14/26, 54%) make less than US $10,000 annually, whereas 100% make less than US $49,999 annually.

### Adolescent Control Participants

Of the control participants, 38% (16/42) are in the adolescent group (aged 14-19 years), with a mean age of 18.8 years (SD 0.5). Among adolescent control participants, 6% (1/16) identify as Black or African American, 25% (4/16) identify as White, and 38% (6/16) identify as Asian. In addition, 50% (8/16) identify as Hispanic or Latinx (categories are not mutually exclusive). All adolescent control participants have received a high school diploma or a GED, and 94% (15/16) are current students. Three-quarters of adolescent control participants make less than US $10,000 annually (12/16, 75%), whereas the remainder makes US $19,999 or less annually.

### Adult Case Participants

Of the participants, 63% (5/8) are in the adult group (aged 20-45 years), with a mean age of 34.6 years (SD 10.4). Among adult case participants, 80% (4/5) identify as Black or African American and 20% (1/5) as White. In addition, 40% (2/5) identify as Hispanic or Latinx (categories are not mutually exclusive). Regarding education, 60% (3/5) of adult case participants have a high school diploma, GED, or less than a high school diploma, whereas 40% (2/5) have a graduate degree, and 20% (1/5) are current students. Most adult case participants (3/5, 60%) make less than US $29,999 annually, whereas 40% (2/5) make US $50,000 or more.

### Adolescent Case Participants

Of the participants, 38% (3/8) are in the adolescent group (aged 14-19 years), with a mean age of 18.3 years (SD 0.6). Among adolescent case participants, 100% (3/3) identify as White and 33% (1/3) identify as Hispanic or Latinx (categories are not mutually exclusive). One (1/3, 33%) of the adolescent case participants has received a high school diploma or GED, and 2 (2/3, 67%) have completed trade or vocational school; 100% (3/3) are current students. Adolescent case participants make less than US $10,000 (2/3, 67%) or US $50,000 or more (1/3, 33%) annually.

## Discussion

The intersecting epidemics of sexual violence and HIV in women is a well-described epidemiologic phenomenon that affects physical and mental health [61,62]. The bidirectional relationship between violence and HIV in women provides a clear but complex target for HIV treatment and prevention. In terms of interventions for violence against women, most approaches focus on perpetration by men [63,64]. HIV prevention efforts in women focus on behavioral interventions that draw from theoretical frameworks of self-efficacy and self-empowerment [65] and on the use of HIV pre-exposure prophylaxis (PrEP) [66]. However, current studies suggest that PrEP failure is more common in women, with multiple factors being delineated, including poor adherence [67-69], sex-based differences in the pharmacokinetics and pharmacodynamics of the antiretrovirals used for PrEP [70], and alteration by the vaginal microbiome [71,72]. Despite evidence that sexual violence is also associated with alterations in the immunobiology of the female reproductive tract, which may increase HIV risk [18], very little is understood about the actual pathogenesis. A clearer understanding of the role of the endocrinologic system in the immunobiology of the female reproductive tract will facilitate the development of novel interventions (both behavioral and pharmaceutical) to enhance PrEP efficacy in women. This study represents the first of its kind to comprehensively evaluate the endocrinologic and local immunobiology of female survivors of sexual violence.

## Abbreviations

ACTH: adrenocorticotropic hormone
CAR: cortisol awakening response
CRP: C-reactive protein
CVL: cervicovaginal lavage
DHEA-S: dehydroepiandrosterone sulfate
FGT: female genital tract
GED: General Educational Diploma
HIPAA: Health Insurance Portability and Accountability Act
HPA: hypothalamic-pituitary-adrenal a
HRPP: Human Research Protection Program
IL: interleukin
MIP3α: macrophage inflammatory protein-3α
PrEP: pre-exposure prophylaxis
SLPI: secretory leukocyte peptidase inhibitor
STI: sexually transmitted infection
T/F: transmitter/founder
THRIVE: Trauma and HIV Risk: Investigating Stress and Immune Disruption of the Vaginal Environment
TNF-α: tumor necrosis factor-alpha
UCSD: University of California, San Diego
